# Catalysis of Chlorovirus Production by the Foraging of *Bursaria truncatella* on *Paramecia bursaria* Containing Endosymbiotic Algae

**DOI:** 10.3390/microorganisms9102170

**Published:** 2021-10-18

**Authors:** Zeina T. Al-Ameeli, Maitham A. Al-Sammak, John P. DeLong, David D. Dunigan, James L. Van Etten

**Affiliations:** 1 Nebraska Center for Virology, University of Nebraska-Lincoln, Lincoln, NE 68583-0900, USA; zeina@huskers.unl.edu (Z.T.A.-A.); s-malsamm1@unl.edu (M.A.A.-S.); ddunigan2@unl.edu (D.D.D.); 2 School of Natural Resources, University of Nebraska-Lincoln, Lincoln, NE 68583-0961, USA; 3 Medical Technical Institutes, Middle Technical University, Bagdad 10047, Iraq; 4 Tropical Biological Researches Unit, College of Science, University of Bagdad, Bagdad 10071, Iraq; 5 School of Biological Sciences, University of Nebraska-Lincoln, Lincoln, NE 68588-2083, USA; jpdelong@unl.edu; 6 Department of Plant Pathology, University of Nebraska-Lincoln, Lincoln, NE 68583-0722, USA

**Keywords:** chloroviruses, *Bursaria truncatella*, *Paramecium bursaria*, symbiosis, ecosystem, predation

## Abstract

Chloroviruses are large viruses that replicate in chlorella-like green algae and normally exist as mutualistic endosymbionts (referred to as zoochlorellae) in protists such as *Paramecium bursaria*. Chlorovirus populations rise and fall in indigenous waters through time; however, the factors involved in these virus fluctuations are still under investigation. Chloroviruses attach to the surface of *P. bursaria* but cannot infect their zoochlorellae hosts because the viruses cannot reach the zoochlorellae as long as they are in the symbiotic phase. Predators of *P. bursaria*, such as copepods and didinia, can bring chloroviruses into contact with zoochlorellae by disrupting the paramecia, which results in an increase in virus titers in microcosm experiments. Here, we report that another predator of *P. bursaria*, *Bursaria truncatella*, can also increase chlorovirus titers. After two days of foraging on *P. bursaria*, *B. truncatella* increased infectious chlorovirus abundance about 20 times above the controls. Shorter term foraging (3 h) resulted in a small increase of chlorovirus titers over the controls and more foraging generated more chloroviruses. Considering that *B. truncatella* does not release viable zoochlorellae either during foraging or through fecal pellets, where zoochlorellae could be infected by chlorovirus, we suggest a third pathway of predator virus catalysis. By engulfing the entire protist and digesting it slowly, virus replication can occur within the predator and some of the virus is passed out through a waste vacuole. These results provide additional support for the hypothesis that predators of *P. bursaria* are important drivers of chlorovirus population sizes and dynamics.

## 1. Introduction

Importance: Chloroviruses are found in most freshwater environments around the world and their titers can fluctuate significantly with the seasons. Their algal hosts typically live as symbionts in other protists such as *Paramecia bursaria*. The chloroviruses can attach to paramecia without infecting them and thus the viruses are present if the paramecia are disrupted and release their symbiotic algae. Previous microcosm studies have demonstrated that organisms (predators) such as copepods and didinia feed on paramecia (prey) by different mechanisms that result in the release of the viral host algae; this release leads to an increase in chlorovirus populations. In the current manuscript, we show that another paramecia predator, *Bursaria truncatella*, with a different feeding behavior, can also cause an increase in chlorovirus titers.

Viruses are the most abundant biological entities on the planet, with ocean water reported to contain ~10^7^ virus particles per mL [1,2,3]. This value translates into ~10^31^ virus particles in the world’s oceans and this estimate does not include RNA viruses or viruses present in soils and other environments. Most of these viruses infect bacteria, with lesser numbers infecting microalgae and other organisms. Consequently, it is becoming clear that viruses and their hosts play major roles in food webs and in the biogeochemical recycling of materials e.g., [4,5].

Chloroviruses (*Phycodnaviridae* family) are large, plaque-forming dsDNA viruses that infect certain chlorella-like green algae that are endosymbionts (referred to as zoochlorellae) in a variety of single and multicellular aquatic organisms [6,7,8]. Chloroviruses are ubiquitous in freshwater habitats including rivers, streams, lakes, marshes, and ponds [9,10,11]. Chlorovirus concentrations can fluctuate seasonally, monthly, or even weekly, with titers occasionally reaching as high as thousands of plaque-forming units (PFU) per mL [12,13].

*Paramecium bursaria* (hereafter referred to as paramecia) is a ciliate found widely in freshwater environments [14] that can harbor several hundred zoochlorellae as endosymbionts, which are hosts for the chloroviruses [15,16,17]. Interestingly, chlorovirus particles can attach to the outside of the paramecia. However, zoochlorellae residing in perialgal vacuoles inside the cytoplasm are protected from chlorovirus infection due to the physical barrier of their host [18,19]. Thus, it has been unclear how chloroviruses replicate in natural environments because access to their algal hosts is limited. Contributing to this issue is the fact that, even though zoochlorellae can be grown in culture in the laboratory [10,11], zoochlorellae do not grow very well, if at all, in indigenous waters free of viruses [Quispe et al., unpublished results]. This observation suggests that free-living zoochlorellae populations are unlikely to grow densities large enough to support abundant chlorovirus populations. However, if zoochlorellae are released from paramecia by either sonication or treatment with a non-ionic detergent (e.g., Triton X-100), the zoochlorellae are readily infected by the viruses and give rise to thousands of infectious viruses per paramecium [20].

Paramecia are prey for a variety of zooplankton such as crustaceans and other protists [19,20]. The act of predation can break the barrier between the zoochlorellae and virus, and can catalyze virus infection. Previous microcosm experiments have demonstrated that predators such as the copepod *Eucyclops agilis* can engulf entire paramecia (referred to as whole-feeding) and release fecal pellets containing viable zoochlorellae that can then be infected by chloroviruses and generate bursts of virus production [20]. In addition, the protist *Didinium nasutum* can disrupt the paramecia by tearing them apart (referred to as messy feeding) and releasing the zoochlorellae into the water where they can be infected [21].

**Figure 1:**
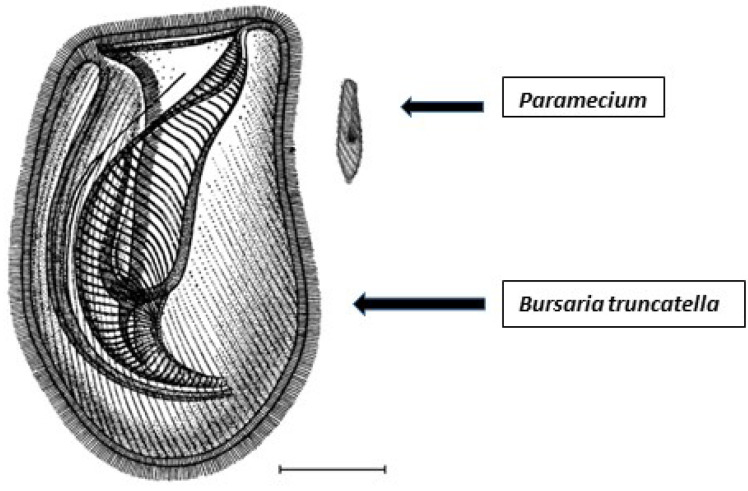
Comparing the size of the predator *Bursaria truncatella* (900–1700 µm) to the size of the prey *Paramecium bursaria* (80–150 µm). Scale bar represents 200 µm. Reprinted with permission from Krause et al., 2009.

The ciliate *Bursaria truncatella* is another predator that feeds on paramecia [22] (Figure 1). However, *B. truncatella* has a different feeding pattern compared to *Eucyclops* and *Didinia* in that it consumes whole ciliates such as paramecia by sweeping them inside their buccal cavity and forming a food vacuole. *B. truncatella* eventually release the waste from a food vacuole through the cell membrane into the surrounding environment [23,24]. In the current manuscript, we examined *B. truncatella* to determine (i) if it also increases chlorovirus concentrations in microcosms by disrupting paramecia and exposing zoochlorellae to chloroviruses during predation, and (ii) the mechanism(s) by which *B. truncatella* could facilitate virus production.

## 2. Materials and Methods

### 2.1. Organisms and Culturing

*B. truncatella* was acquired from Carolina Biological Supply (CBS; Burlington, North Carolina) and cultured in CBS spring water at 23 °C. *P. bursaria* were collected from a pond at Spring Creek Prairie Audubon Center, near Denton, NE, USA (GPS coordinates: 40°41′37.6764″ N, 96°51′12.2544″ W) [25]. Paramecia were maintained at 23 °C in CBS spring water with constant light (light flux, 38 to 42 µmol/m^2^ s^−1^). *B. truncatella* was maintained in the laboratory using a mixture of paramecia culture and a standardized hay–wheat medium, prepared by mixing 5 g of dry hay with 5 g of wheat grain in 2 L of autoclaved water collected from Spring Creek. The mixture was boiled for 5 min and filtered through a Whatman filter paper 4 (pore size 20–25 μm, circle size 11 cm, Whatman Scheicher & Schuell, Whatman International Ltd. Maidstone, England). Sterile pond water from Spring Creek was produced by a series of filtration steps. Pond water was first filtered with Whatman filter paper 4 (pore size 20–25 µm) to remove large debris, followed by subsequent filtration (pore size 0.45 µm). Finally, the pond water was filtered (0.1 µm) and autoclaved at 121 °C for 60 min. Protozoan medium was also prepared by mixing 100 mL of CBS spring water with 900 mL of autoclaved pond water from Spring Creek.

*B. truncatella* and paramecia interactions, and their impact on chlorovirus (strain Osy-NE-ZA1) titers were monitored in two types of experiments. Long (48 h) and short-term (3 h) foraging experiments were conducted in 3 mL of microcosms in Petri dishes (35 mm diameter) with lids. In all of the foraging experiments, the *B. truncatella* were removed from growing stock cultures, rinsed three times with sterile pond water, and then placed in sterile pond water for one day prior to the start of the experiments to ensure that their buccal cavity and insides were apparently free of nutrients [20].

### 2.2. Long-Term Foraging Experiments

The microcosms for the long-term foraging experiments consisted of 30 paramecia in 1 mL of culture media, 1 mL of protozoan media, and 1 mL of hay–wheat media containing one *B. truncatella* for a total of 3 mL. Three treatments were created: (i) five replicates of microcosms with 30 paramecia only (negative control); (ii) five replicates of microcosms where the 30 paramecia were sonicated at the beginning of the experiment using a Tekmar Sonic Disruptor (model number TM100) for ~10 s at an output level of 5 to disrupt the paramecia and release the algae (positive control); and (iii) ten replicates of microcosms with one *B. truncatella* and 30 paramecia. The *B. truncatella* were allowed to feed on the paramecia for 48 h. Each day, 0.5 mL of water was removed from the microcosm cultures, ensuring that none of the paramecia or *B. truncatella* were picked up as well, and replaced with 0.5 mL of autoclaved pond water. Infectious viral titers in all of the experiments were monitored daily from these samples by triplicate plaque assays on lawns of *Chlorella variabilis* Syngen 2-3 [26]. Samples were first passed through a 0.45 µL filter. *B. truncatella* and paramecia from each microcosm were counted daily at the time of sampling. All trials were performed at 23 °C with constant light.

### 2.3. Short-Term Foraging Experiments

**Figure 2:**
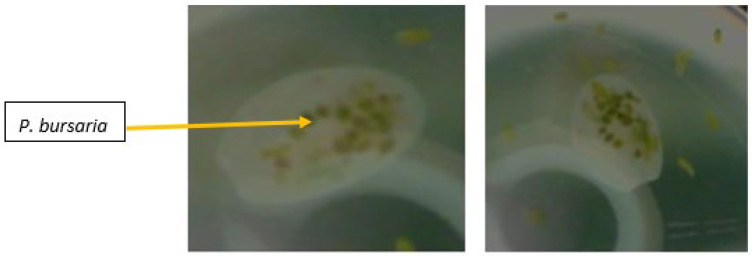
Images of *B. truncatella* after foraging on paramecia for 48 h in a microcosm. Note the many paramecia inside the *B. truncatella*.

In the short-term foraging experiments, three treatments were created, consisting of 30 paramecia in 2 mL of protozoa medium and 1 mL of hay–wheat medium containing one *B. truncatella*: (i) 30 paramecia only (negative control, 5 replicates); (ii) 30 sonicated (~10 s) paramecia (positive control, 5 replicates); and (iii) 30 paramecia with one *B. truncatella* (18 replicates). The *B. truncatella* were allowed to forage on paramecia for ~3 h. During this time, food vacuoles formed inside the *B. truncatella*. After 3 h, the *B. truncatella* were removed, rinsed 3 times with sterile pond water, and placed in Petri dishes in 2 mL of sterile pond water. The *B. truncatella* were transparent and the green paramecia could be seen inside the cell (Figure 2), which allowed us to count the number of ingested paramecia. We used the ingested number as a direct measure of foraging. Water samples (0.5 mL) were removed from the microcosm with the rinsed *B. truncatella* at 0, 24, and 48 h, and 0.5 mL of sterile pond water was added back to the Petri dishes after each sampling. Virus abundances were assessed by plaque assays.

### 2.4. Monitoring Chloroviruses Inside B. truncatella

A separate set of paramecia were chemically disrupted using 0.5% Triton X-100 to determine the number of PFUs attached to the outside of the paramecia using the plaque assay. Unwashed paramecia released 200–600 PFU/individual. After washing with sterile pond water three times, the paramecia contained 100–150 PFU per paramecium. This confirms that *B. truncatella* consumes the virus along with paramecia during the foraging, which opens up the possibility that *B. truncatella* could pass viable viruses through their vacuoles post-digestion. We therefore conducted a separate experiment to check if the virus replicated inside the food vacuoles or whether *B. truncatella* simply released the viruses that were attached to paramecia at the time they were consumed. First, we rinsed *B. truncatella* with sterile pond water three times and placed them in Petri dishes containing 3 mL of sterile pond water for one day. This ensured that their buccal cavity was free of any food. After that, we created twelve 2 mL Petri dish microcosms with 30 paramecia and one *B. truncatella* in each microcosm. The *B. truncatella* were allowed to feed on paramecia for ~3 h. *B. truncatella* that had consumed three paramecia were removed, rinsed three times with sterile pond water, and put in 1 mL of sterile pond water. Individual microcosms of *B. truncatella* were exposed to 20–40 µL of 0.5% Triton X-100. Normally it requires about 6.5 h for the virus to replicate [9] and if the virus was replicating inside the food vacuoles, we expected that chlorovirus titers would increase around 6.5 h. Otherwise, the virus titers within *B. truncatella* would either decline or remain constant. Plaque assays were conducted for each sample. *T*-tests and linear regression analyses were conducted in MS Excel.

## 3. Results

### 3.1. Long-Term Foraging Experiments

**Figure 3:**
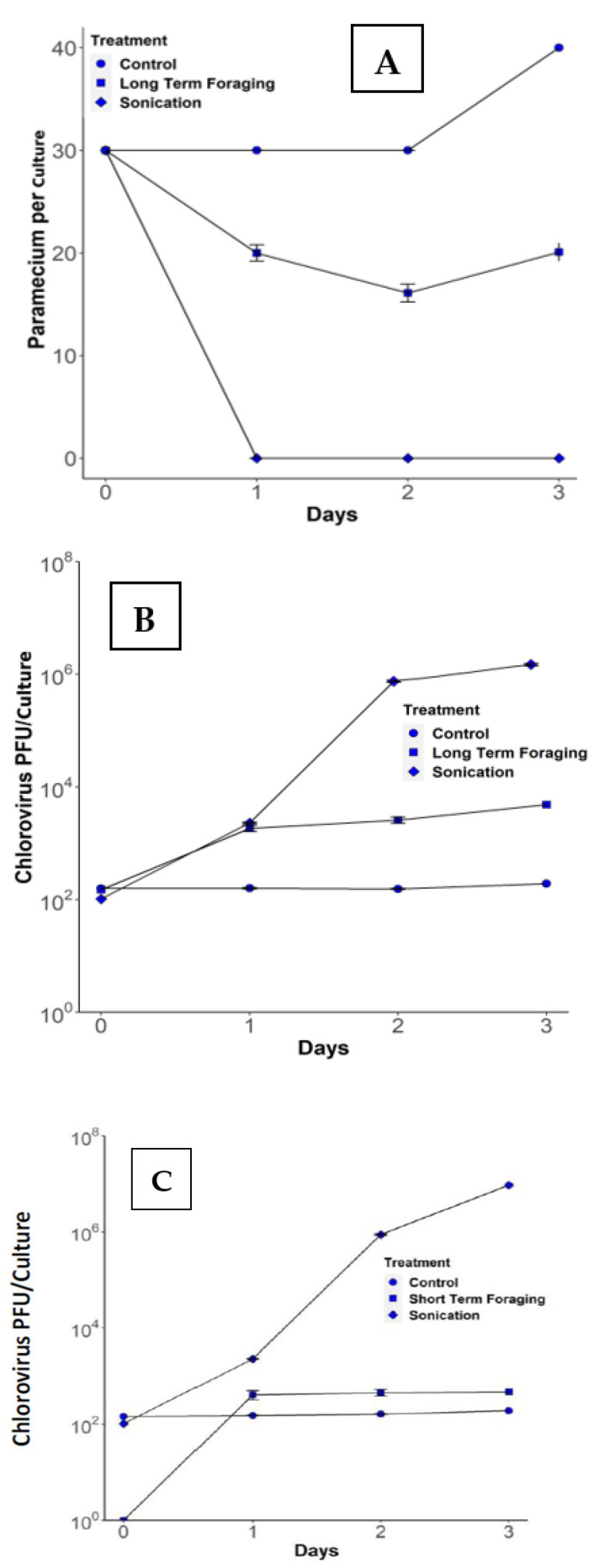
Paramecium and chlorovirus dynamics in a long-term foraging experiment. (**A**) Paramecia abundance decreased in foraging compared to the control and sonication treatments. (**B**) Long-term foraging (48 h) results in an ~20-fold increase in chlorovirus’s PFU/culture compared to the control. (**C**). Short-term foraging by *B. truncatella* for seven of the eighteen replicates. Short-term foraging produced an increase in chlorovirus concentrations after 24 h compared to the control, which remained constant, and in sonication, which represents the positive control.

The initial experiments were designed to determine if *B. truncatella* feeding on paramecia catalyzed an increase in chlorovirus titers. In the control dishes, paramecium abundance was stable and then increased by day 3 (Figure 3A), while chlorovirus titers remained constant throughout the experiment at 150 ± 10 PFU/culture (Figure 3B). In the sonication treatment, all paramecia were disrupted (Figure 3A) and chlorovirus titers increased steadily to 8.8 × 10^5^ ± 6.7 × 10^4^ PFU/culture by day 2 (Figure 3B). In the foraging treatments, the number of paramecia decreased (Figure 3A) and the chlorovirus population increased with time to 2900 ± 1313 PFU/culture (Figure 3B). Thus, in the presence of *B. truncatella*, the number of chloroviruses increased about 20 times from ~150 PFU/culture to 2900 PFU/culture at 48 h (*t* = −7.01, *p* < 0.001, d.f. = 18; Figure 3B). However, the 2900 PFU/culture of chloroviruses produced by *B. truncatella* feeding at 48 h was only about ~0.3% of the amount produced at 48 h in the sonication treatments. Virus titers did not increase with the number of paramecia consumed during the experiment (*t* = 1.74, *p* < 0.099, *n* = 10). A possible explanation is that as the predators consume paramecia, there is a feature that the *B. truncatella* possess which stops them from consuming more paramecia than they can handle until they digest all the contents in their digestive tract. As more paramecia are consumed, a large mass of paramecia accumulates inside the food vacuole, which results in the *B. truncatella* taking a longer time to digest the contents inside the food vacuole. Presumably, *B. truncatella* releases digestive enzymes inside the food vacuole, which can inactivate the virus particles and the zoochlorellae. This leads to a negative effect on vital algae and virus particles, resulting in low virus concentrations being released into the microcosm. In short-term foraging, the *B. truncatella* consume a small number of paramecia, resulting in the faster digestion of the paramecia inside the food vacuole. This results in more algal cells and virus particles surviving and being released into the microcosm.

### 3.2. Short-Term Foraging Experiments

In the short-term experiment, seven of the eighteen microcosms showed foraging along with slight increases in chlorovirus populations, while the remaining eleven microcosms had no foraging and no increase in chlorovirus populations. The *B. truncatella* in the seven microcosms that consumed paramecia showed an increase in chlorovirus titers of about ~300 PFU/culture (Figure 3C). After 24 h, there were 457 ± 193 PFU/culture and the number increased to slightly over 457 PFU after 48 h. The *B. truncatella* that ate more paramecia generated higher levels of PFUs in the dishes (*t* = 5.70, *p* = 0.002, R^2^ = 0.86).

### 3.3. Disruption of the B. truncatella

**Figure 4:**
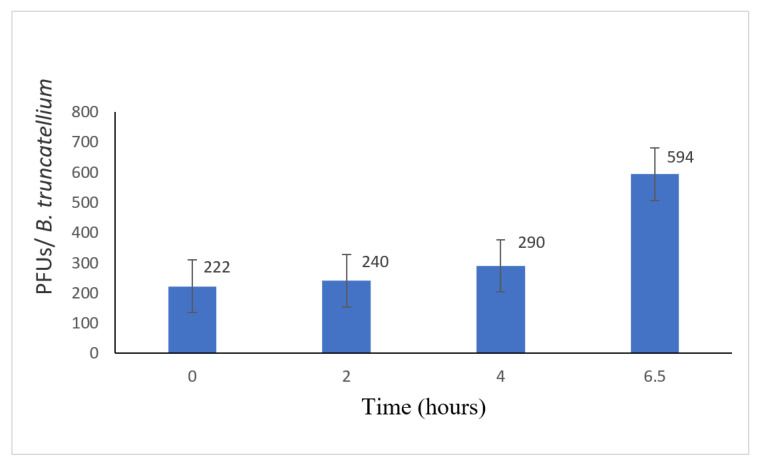
Chlorovirus replication inside *B. truncatella* at four times (PFU/*B truncatella*). Chlorovirus titers remained about the same up until 4 h. After 4 h, the PFU of the chloroviruses increased.

As the number of PFU was low, i.e., ~230 PFU per paramecium consumed, it was possible that virus replication was not occurring and the *B. truncatella* were simply stripping the infectious chloroviruses attached to the surface of the paramecia. To investigate this possibility, we measured virus titers inside the *B. truncatella* by disrupting them with Triton at 0, 2, 4, and 6.5 h after the *B. truncatella* had consumed the paramecia. Virus titers of the Triton-treated *B. truncatella* remained reasonably constant at 0, 2, and 4 h, but then doubled at 6.5 h, suggesting that the viruses were replicating inside the *B. truncatella*, as this is about how long the viruses require to begin replicating (Figure 4) [27]. This finding indicates that at least some of the increase in the virus titers during the experiment was due to virus replication within the food vacuole while the zoochlorellae were still viable and not solely due to the release of virus particles that were initially attached to the paramecia.

## 4. Discussion

Chloroviruses are ubiquitous in freshwater environments, and virus concentrations can fluctuate weekly, with titers occasionally reaching thousands of PFU/mL of water (11–13). An ongoing question has been: how do the chloroviruses replicate in their natural environments? This question is relevant because zoochlorellae are protected from chlorovirus infection while they are in their symbiotic phase due to the physical barrier of the paramecium cell membrane, which prevents chlorovirus–zoochlorellae interactions. There is no evidence for an alternative chlorovirus host and, although zoochlorellae can grow in culture in the laboratory, the zoochlorellae grow poorly, if at all, in native waters free of viruses (Quispe et al., unpublished results). It is known, however, that chloroviruses can attach to the external surface of paramecia without infecting them [17,18,19]. Consequently, the viruses are positioned to attack the zoochlorellae if the paramecia are disrupted.

Some understanding of this dilemma has been provided by two recent reports of predator catalysis. Predators of paramecia, such as the copepod *Eucyclops aeglis*, can engulf entire paramecia (referred to as ‘whole-feeding’) and release fecal pellets containing viable zoochlorellae, which can then be infected by chloroviruses and generate an ~100-fold increase in chlorovirus titers (20). In a similar manner, the protist *Didinium nasutum*, a predator of paramecia, can disrupt paramecia by tearing them apart (referred to as ‘messy feeding’) and releasing zoochlorellae into the water where they can be infected, leading to an ~1000-fold increase in virus titers [21]. Larger *Didinium* can engulf the entire paramecia (whole-feeding) and, when they do so, stimulate less virus production than smaller *Didinia*.

The current manuscript provides evidence that another ciliate, *B. truncatella*, which has a different feeding pattern than both *E. agilis* and *D. nasutum*, also increases chlorovirus titers by about 20-fold (Figure 3). *B. truncatella* consume the entire paramecia by sweeping them inside the buccal cavity where they form a food vacuole [23,24,25,28,29]. The food vacuoles are easily distinguished from other vacuoles because of the green color from the engulfed zoochlorellae. The paramecia are degraded in the food vacuoles and the *B. truncatella* eventually release the waste content through the posterior cytopyge [23,24,25]. This waste likely includes some ‘viable’ virus, as indicated in our short-term experiments, because the *B. truncatella* were rinsed and transferred to clean dishes, leaving no other opportunity for viruses to increase. This scenario suggests that there is a latency post-ingestion in which viral replication can occur within the *B. truncatella*’s food vacuole before they are digested and some of these viruses survive digestion and are released into the microcosm. Alternatively, the increases in PFU could be due to several other factors, one of which is that the chloroviruses attached to the paramecia’s cell membrane are released into the water upon consumption. However, even if this were the case, the number would have remained constant throughout the 48 h rather than increase (Figure 4) because there would be no replication occurring. Another alternative is that some of the zoochlorellae could possibly be discharged into the microcosm through messy feeding, leading to virus replication, but this is not likely a major cause, as this was only observed once during the many times we conducted these experiments.

**Figure 5:**
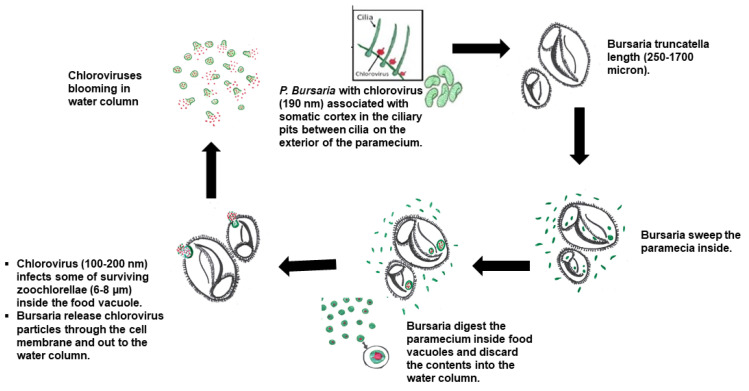
Sequence of events leading to an increase in chlorovirus populations by the predator *B. truncatella*. Chlorovirus particles attach to the outside of the paramecia’s cell membrane (red particles) and paramecia host the symbiotic zoochlorellae (green), which are the host for the chloroviruses. *B. truncatella* engulf the entire paramecium. Inside the food vacuole, chloroviruses infect some of the released zoochlorellae, leading to viral replication. After a few hours, the *B. truncatella* release the waste material through the membrane into the environment and the waste material contains in-fectious virus particles.

Three different types of organisms have been described that can feed on paramecia and consequently disrupt the barrier between the chloroviruses and their host zoochlorellae [20,21]. All three predators, which may be present at the same time in nature, probably contribute to the size of chlorovirus populations. However, judging from the microcosm experiments, they are not equally efficient. For example, *Didinium* generated ~95% of its theoretical yield (as judged by sonication) of virus production compared to the ~17% of the theoretical yield for the copepods [20,21]. In the case of *B. truncatella*, the number of chloroviruses generated was only ~0.3% of the maximum yield (Figure 5). The *B. truncatella’s* feeding process in the microcosms was also slower than the two other predators we examined, as a food vacuole can exist in *B. trucatella* for up to 7 h, providing enough time for virus replication to occur, as reported in Figure 4. Digestive enzymes help bursaria to disrupt the paramecia cell membrane and inactivate some of the viral particles and host cell. Nonetheless, some of them still survive, which helps the virus in continuing to replicate [20,21]. These three predator catalyst mechanisms differ from other predator effects on parasite and disease transmission, such as concerning the promotion of epidemics in *Daphnia*, where predators release parasitic fungal spores from infected *Daphnia* prey into the environment [29,30] or when predators defecate virus particles into new areas after they consume infected prey [31,32].

The predator–paramecia–zoochlorellae–chlorovirus relationships are part of larger aquatic food webs. In addition to copepods, *Didinium*, and *B. truncatella*, other aquatic predators, including nematodes, *Stentor*, and planarians, are reported to feed on paramecia [20,21,32,33,34,35,36] and could influence both chlorovirus populations and the microbial food web [35,36]. One would predict, then, that the composition of the predator community feeding on paramecia would influence the size of chlorovirus populations and their tendency to cycle in nature [36]. Furthermore, predators such as larger zooplankton and fish could have a trophic-cascade-like effect on virus activation because they also may consume copepods, *Didinium*, and/or *B. truncatella*, but the role those larger predators play on chlorovirus titers in natural environments, if any, is unknown.

## 5. Conclusions

Microcosm experiments establish that *B. truncatella* can disrupt paramecia carrying symbiotic zoochlorellae. There is a period post-ingestion in which chloroviruses replicate inside the food vacuole. We suggest that some of these viruses survive digestion and are then released into the microcosm. This interaction results in slight increases in chlorovirus populations and can help explain the consistent presence of chlorovirus titers in nature.

## Data Availability

Not applicable.
